# Barriers to essential surgical care experienced by women in the two northernmost regions of Ghana: a cross-sectional survey

**DOI:** 10.1186/s12905-016-0308-4

**Published:** 2016-05-26

**Authors:** Adam Gyedu, Francis Abantanga, Godfred Boakye, Shailvi Gupta, Easmon Otupiri, Anita Eseenam Agbeko, Adam Kushner, Barclay Stewart

**Affiliations:** Department of Surgery, School of Medical Sciences, Kwame Nkrumah University of Science and Technology, Kumasi, Ghana; Directorate of Surgery, Komfo Anokye Teaching Hospital, Kumasi, Ghana; School of Public Health, Kwame Nkrumah University of Science and Technology, Kumasi, Ghana; Department of Surgery, University of California, San Francisco, CA USA; Surgeons OverSeas (SOS), New York, NY USA; Department of International Health, Johns Hopkins Bloomberg School of Public Health, Baltimore, MD USA; Department of Surgery, Columbia University, New York, NY USA; Department of Surgery, University of Washington, Seattle, WA USA

**Keywords:** Barriers, Women, Essential surgical care, Ghana, LMIC

## Abstract

**Background:**

Women in developing countries might experience certain barriers to care more frequently than men. We aimed to describe barriers to essential surgical care that women face in five communities in Ghana.

**Methods:**

Questions regarding potential barriers were asked during surgical outreaches to five communities in the northernmost regions of Ghana. Responses were scored in three dimensions from 0 to 18 (i.e., ‘acceptability,’ ‘affordability,’ and ‘accessibility’; 18 implied no barriers). A barrier to care index out of 10 was derived (10 implied no barriers). An open-ended question to elicit gender-specific barriers was also asked.

**Results:**

Of the 320 participants approached, 315 responded (response rate 98 %); 149 were women (47 %). Women had a slightly lower barriers to surgical care index (median index 7.4; IQR 3.9–9.1) than men (7.9; IQR 3.9–9.4; *p* = 0.002). Compared with men, women had lower accessibility and acceptability dimension scores (14.4/18 vs 14.4/18; *p* = 0.001 and 13.5/18 vs 14/18; *p* = 0.05, respectively), but similar affordability scores (13.5/18 vs 13.5/18; *p* = 0.13). Factors contributing to low dimension scores among women included fear of anesthesia, lack of social support, and difficulty navigating healthcare, as well as lack of hospital privacy and confidentiality.

**Conclusion:**

Women had a slightly lower barriers to surgical care index than men, which may indicate greater barriers to surgical care. However, the actual significance of this difference is not yet known. Community-level education regarding the safety and benefits of essential surgical care is needed. Additionally, healthcare facilities must ensure a private and confidential care environment. These interventions might ameliorate some barriers to essential surgical care for women in Ghana, as well as other LMICs more broadly.

**Electronic supplementary material:**

The online version of this article (doi:10.1186/s12905-016-0308-4) contains supplementary material, which is available to authorized users.

## Background

Conditions that benefit from timely, safe surgery comprise nearly 16 % of the global disease burden [1]. However, up to five billion people, most of whom live in low- and middle-income countries (LMICs), do not have access to essential surgical care as defined by the world bank’s Disease Control Priorities, third edition (DCP-3) [2]. Essential surgical care consists of surgical decision-making and procedures that address high-burden conditions, are cost effective, and are feasible to implement. Given this gap between the burden of surgical conditions and the availability of surgical care services, LMICs have high prevalence of unmet surgical need - a situation where a person has a condition treatable by essential surgical care or is in need of a surgical consultation and is not able to access required care [3, 4]. This is true even for common conditions (i.e. skin and soft tissue masses, breast and gynecologic problems) [3, 5].

The most significant barrier to surgical care in LMICs is inadequate capacity (i.e., infrastructure, human, and physical resources) [6]. While urgently needed, sufficient investment in global surgical care is unlikely to occur in the short term. [7] Therefore, in addition to supporting surgical care development, communities and countries must identify and remove other barriers to surgical care that their populations face in order to significantly reduce the surgical disease burden.

Certain sub-populations, such as women, might be particularly vulnerable to certain barriers that are not significant obstacles for the rest of the population [8]. Therefore, targeted interventions that ameliorate specific barriers may be needed for such sub-populations within a community. Identifying and removing such barriers often represents a cost-effective method for improving the uptake of essential surgery for groups that might have otherwise been excluded from this vital service.

Grimes et al. performed a systematic review of reports that described barriers to surgical care in LMICs [9]. They usefully sorted the barriers identified by the review into 20 themes that represent 3 dimensions: acceptability, affordability and accessibility. However, the review did not return a report that described barriers to essential general surgical care among women in LMICs.

To address this gap, we used this framework to develop a comprehensive tool to assess individual- and community-level barriers to surgical care in LMICs such as Ghana [10]. For this study, we aimed to describe the barriers to surgical care that women face in five particularly deprived communities in northern Ghana and compare them to the barriers faced by men. By doing so, gender-specific barriers could be identified and potential targets for intervention defined.

## Methods

### Assessment tool

By using a modified Delphi technique we developed the assessment tool by creating questions to represent each of the barriers to surgical care identified by the systematic review by Grimes et al. [9]. Modifications to the Delphi technique included the use of a panel of 5 experts (with experience in developing community-based surveys for LMICs) and not using quantitative methods to include or exclude specific questions for the subsequent round. Instead, the panelists were only given the option of including or excluding the question in the next round, as well as offering questions for the group to evaluate in successive rounds. An initial exploration of potential questions and three survey rounds were used to build consensus on questions that were thought to accurately represent each theme, be relevant for LMICs and be simple to administer. The resultant list of questions was sorted into themes and grouped into the three dimensions reported by the Grimes et al. review:

- Acceptability – fear and/or mistrust of surgery; marginalized social status; reduced appreciation of medical conditions; degree of their impairment
- Affordability – high direct and/or indirect costs of essential surgical care; lack of social support
- Accessibility – delay in diagnosis; healthcare navigation; structural (i.e., distance and road quality)

Ultimately, 38 barrier-specific questions were included in the tool - four aimed to identify potentially vulnerable sub-populations; 21 represented acceptability; 9 represented accessibility; and 4 represented affordability (Additional file 1). Prior to asking about specific barriers to care, respondents were prompted with: “Which of the following reasons for not having surgery for your problem sooner apply to you?”

At the end of the structured portion of the tool, each patient was prompted to consider and discuss other reasons for not receiving timely surgical care that were not captured by prior questions. Additionally, an open-ended question that aimed to elicit gender-specific barriers not captured by the quantitative section of the tool was included.

### Calculating dimension scores and the overall index

Individual dimension scores (i.e., acceptability, affordability, accessibility) were calculated by adding one point for each item that was not a barrier to care. The sum was then multiplied by a dimension factor so that each dimension could have an equal total possible score of 18 points, which would allow apposite comparison. Next, dimension scores were added together. Lastly, the sum of the dimension scores was indexed on a scale from zero to 10, where 10 represented no barriers to surgical care. The resulting index was termed the barriers to surgical care index. Similar indexing methods have been successfully used for surveys of surgical capacity in LMICs [3, 11].

### Setting

During a surgical outreach by ApriDec Medical Outreach Group (AMOG), the tool was administered in five communities chosen to represent populations from particularly deprived areas in the northernmost regions (i.e., Upper East and Upper West Regions) of Ghana. AMOG is a Ghanaian-based non-governmental organization (NGO) that performs free surgical outreach after intensive mobilization in areas where significant barriers to surgical care may exist. Sites were purposely sampled to represent rural, peri-urban and urban populations and the regional diversity of surgical capacity. The sites were Nadowli, Nandom, Sandema, Amiah and Bolgatanga. Nadowli, Nandom and Sandema are rural districts with large catchment areas, no general surgeon and significant resource-deficiencies with regards to surgical care capacity. Amiah and Bolgatanga are peri-urban and urban respectively; have a general surgeon each, but are strained by high demand. Surgical care in each of the sampled sites suffers from baseline physical and human resource deficiencies previously documented in Ghana [12, 13].

### Patient sampling and data collection

Community leaders, radio announcements and visually informative flyers in the respective local languages at social activity areas (e.g., churches, mosques, markets) were used to mobilize patients for surgical evaluation several weeks prior to the visit of the outreach team. Patients who presented for evaluation were examined and registered by the hospital staff if they had a potential surgical condition and asked to return during outreach dates.

All patients who presented for surgery were exhaustively sampled. The number of respondents was limited by the effectiveness of the mobilization techniques and the number of operations that the volunteers could perform during the outreach period (i.e., 5 days at each site). Thus, the calculation of a pre-determined sample size and sampling strategy that accurately represented each community was not performed.

At each site, nurses or medical assistants who lived in the respective community and spoke at least one of the local languages were trained as research team members prior to the outreach to ensure adequate skills for conducting interviews and limiting potential interview bias. Team member training focused on interviewing techniques (e.g., questionnaire administration, managing the interview environment and process, active listening, open questioning, reorientation, probing techniques, and response scoring, and ethics) over two days. The tool was translated from English to each of the required local languages and back translated into English to ensure validity of verbal translation by each research team member. After, the assessment tool was verbally administered to each patient in his or her primary language prior to pre-operative preparation to avoid perception contamination that might occur after receiving surgical care (i.e., changing one’s mind about surgical care after having received an operation). Women were able to choose between a male and a female interviewer to minimize interviewer-induced bias.

### Data analysis

First, the internal consistency and construct validity of the tool were assessed using Cronbach’s alpha and confirmatory factor analysis (Additional file 1). For the latter, both the root mean square error of approximation (RMSEA) and coefficient of determination (CD) were calculated.

Second, individual dimension scores and the total barrier to care index for both genders were calculated using Stata v13 (College Station, TX, USA). The Wilcoxon Mann-Whitney test was used to determine whether there was a difference between the barriers to care indices for the two genders. Next, bivariate and three-level mixed effects multivariable logistic regression analysis were performed to determine the effect of being a woman on the odds of having an index in the lowest quartile. The multivariable model included each of the *a priori* defined potentially vulnerable sub-population covariates (i.e., women, children aged less than 18 years, older adults aged more than 50 years, non-literacy, and religious minority). Minors and the elderly were grouped together because of low numbers. The mixed effects model included covariates for region and community to control for intra-class correlation. There was no evidence for significant multicollinearity among the covariates in the model (1.01 ≤ variance inflation factor ≤1.04). The regression analysis was also performed strictly among women.

Lastly, responses to the qualitative question were analyzed using a content analysis framework [14]. First, responses were grouped into coded categories that represented similar responses. Then, categories were refined into useful themes and described.

### Ethics

The Kwame Nkrumah University of Science and Technology Committee for Human Research and Publication Ethics (reference number – CHRPE/AP/391/14), leadership of AMOG, the Regional Health Directorate of the Ghana Health Service and administration of each facility approved the study.

Adults underwent verbal informed consent in the patient’s primary language. During the consent process, patients were made aware that participation in the survey had no bearing on their eligibility to receive surgical care. For patients aged less than 18 years, an adult relative supervising the child’s hospital stay provided informed consent. Since a child’s access to care is dependent on the parents’ or guardian’s perceptions and means, questions were directed to that person during the assessment, as opposed to the child [15].

## Results

### Internal consistency and construct validity

Overall, Cronbach’s alpha was 0.72, which represents a reasonable degree of internal consistency. Cronbach’s alpha values for each of the dimensions demonstrated only moderate reliability: acceptability 0.69; affordability 0.53; accessibility 0.43. Note that the modest values may reflect a lack of inter-relatedness of the barriers to care within each dimension (e.g. fear of surgery and the degree of symptoms causing impairment of daily work are both listed with the acceptability dimension).

Confirmatory factor analysis revealed evidence for there being a correlation between each question and the dimensions they were supposed to represent, as well as between each dimension and the total barrier index (Additional file 1). The RMSE for the index model was 0.001; the probability of the RMSEA being ≤0.05 was 1.00. The index model coefficient of variance was 0.76, which demonstrates reasonable model fit. It should be noted that the number of respondents was smaller than that recommended for a robust confirmatory factor analysis [16]; nonetheless, the results suggest reasonable construct validity.

### Demographics and operations

The assessment tool was administered to 310 of the 315 participants approached at the five sites (response rate 98 %). One person refused to participate and four were operated on before being interviewed. The median age was 40 years (range 1–81 years). There were 149 women (47 % of respondents). Most women had no formal education (84, 56 %); median travel time to health facilities was 60 min (IQR 0.2–8 h). There was no evidence for a difference in age, education level, and travel time to the health facility between women and men. However, women had a longer median duration of surgical condition (48 months; IQR 1–240 months) compared to men (36 months; IQR 2–360; *p* = 0.02). Both women and men most commonly practiced Christianity (77 % and 54 %, respectively). However, more men practiced a traditional religion when compared with women (33 % vs 6 %; *p* < 0.001). Women most commonly presented with a gynecologic problem (54 %) or a goiter (17 %); men most commonly presented with a hernia or hydrocele (75 %) (Table 1).

**Table 1:** Participant demographic information and operations performed at northernmost regions of Ghana

	Women		Men		Total		
	*n*	(%)	*n*	(%)	*n*	(%)	*p*-value
Participants	149	(47)	166	(53)	315	(100)	
Age; median (IQR)	38	(16–71)	42	(1–80)	40	(1–80)	0.40
Education completed							
None	84	(56)	102	(62)	187	(59)	0.11
Primary	27	(18)	40	(24)	68	(22)	
Secondary	24	(16)	14	(8)	38	(12)	
More	16	(10)	9	(6)	23	(7)	
Religion							
Christian	114	(77)	88	(54)	204	(65)	<0.001
Traditional	9	(6)	54	(33)	63	(20)	
Muslim	24	(16)	19	(12)	43	(14)	
Other	1	(1)	3	(2)	4	(1)	
Travel time; median min (IQR)	60	(10–480)	60	(5–510)	60	(5–1,441)	0.45
Conditions							
Hernia/hydrocele	18	(12)	124	(75)	142	(45)	<0.001
Goiter	25	(17)	1	(1)	26	(8)	
Skin/soft tissue mass	19	(13)	14	(8)	35	(11)	
Gynecologic problem	80	(54)	8	(5)	88	(28)	
Other	6	(4)	18	(11)	24	(8)	
Duration of problem, median months (IQR)	48	(1–240)	36	(2–360)	36	(1–360)	0.02

*IQR* interquartile range, *mins* minutes

### Barrier dimension scores and index

**Fig. 1:**
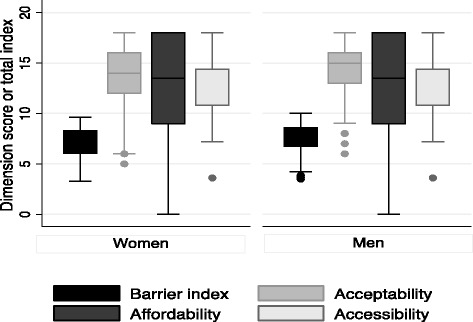
Box-plot of barriers to surgical care index and individual dimension scores by gender in the northernmost regions of Ghana

Women had a statistically significantly lower median barrier to surgical care index (7.4; IQR 3.9–9.1) compared with men (7.9; IQR 3.9–9.4; *p* = 0.002). There was no evidence for a difference in the affordability dimension score between men and women (13.5 out of 18 for both, respectively; *p* = 0.13). However, women had a lower median acceptability dimension score (14.0 out of 18) than men (15.0 out of 18) (*p* = 0.05). Similarly, there was evidence for a difference in the accessibility dimension score between the genders (14.4 out of 18 for both men and women; *p* = 001) (Table 2). Although both genders had equal median accessibility scores (14.4 out of 18), women had a lower rank sum value (21,136) than men (28,634) giving large differences in the IQR (women 3.6–18 vs men 7.2–18), implying that they had lower scores (Fig. 1).

**Table 2:** Barriers to surgical care index and individual dimension scores by gender in the northernmost regions of Ghana

	Total		Women		Men		*p*-value
	Median	(IQR)	Median	(IQR)	Median	(IQR)	
BSC Index (out of 10)	7.7	(3.6–9.4)	7.4	(3.9–9.1)	7.9	(3.9–9.4)	0.002
Acceptability (out of 18)	14.0	(6.0–18.0)	14.0	7.0–18.0)	15.0	(8.0–18.0)	0.05
Affordability (out of 18)	13.5	(0.0–18.0)	13.5	(0.0–18.0)	13.5	(0.0–18.0)	0.13
Accessibility (out of 18)	14.4	(3.6–18.0)	14.4	(3.6–18.0)	14.4	(7.2–18.0)	0.001

*BSC* barriers to surgical care, *IQR* interquartile range

There was evidence for women having greater odds of a barrier to care index in the lowest quartile in the bivariate regression model; however, this was not demonstrated by the multivariable model (adjusted OR 1.32; 95%CI 0.72–2.43) (Table 3). Among female respondents only, minors and the elderly and those with no formal education had higher odds of having a barrier to care index in the lowest quartile in both the bivariate or multivariable regression models; however the increase in odds was not statistically significant (Table 4).

**Table 3:** Odds ratios of having a barrier to care index in the lowest quartile (i.e., most significant barriers to surgical care) in northernmost regions of Ghana

	Odds ratio	(95 % CI)	Adj. odds ratio	(95 % CI)
Sex				
Men	Referent		Referent	
Women	1.92	(1.15–3.24)	1.32	(0.72–2.43)
Age				
18–50 years	Referent		Referent	
Extreme ages^a^	0.75	(0.44–1.28)	0.86	(0.48–1.56)
Education				
None	Referent		Referent	
Any	1.17	(0.69–1.98)	1.23	(0.70–2.18)
Religion				
Christian	Referent		Referent	
Religious minority	0.70	(0.40–1.21)	0.91	(0.49–1.69)

*Adj. odds ratio* adjusted odds ratio; the multivariate model included each covariate given their a priori potential for representing vulnerable sub-populations, as well as community to control for intra-class correlation. Proportional change in variance = 77 %

^a^Extreme ages: <18 years or >50 years

**Table 4:** Factors affecting a barrier to care index in the lowest quartile (i.e. most significant barriers to surgical care) among women in the northernmost regions of Ghana

	Odds ratio	(95 % CI)	Adj. odds ratio	(95 % CI)
Age				
19–50 years	Referent		Referent	
Extreme ages^a^	1.24	(0.58–2.67)	1.37	(0.57–3.26)
Education				
Any	Referent		Referent	
None	1.38	(0.68–2.79)	1.50	(0.721–3.17)
Religion				
Christian	Referent		Referent	
Religious minority	0.72	(0.31–1.69)	0.77	(0.31–1.87)

*Adj. odds ratio* adjusted odds ratio; the multivariate model included each covariate given their a priori potential for representing vulnerable sub-populations, as well as community to control for intra-class correlation. Proportional change in variance = 42 %

^a^Extreme ages: <18 years or >50 years

### Factors contributing to low dimension scores

**Fig. 2:**
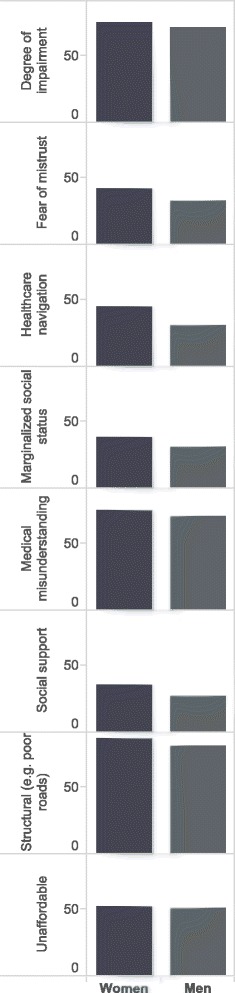
Relative proportions of barriers to care themes by gender in the northernmost regions of Ghana

Figure 2 demonstrates the reported barriers to surgical care by theme. Women more often reported barriers than men for all themes; some examples are worth mentioning. More women reported difficulty in navigating healthcare system compared with men (67; 45 % vs 51; 31 %; *p* = 0.04). Sixty-two women (42 %) reported fear or mistrust of surgical care and 56 (38 %) reported that they felt socially marginalized or were afraid of social stigma regarding their surgical condition. Among men, these barriers were less frequently reported (54; 33 % and 52; 31 %, respectively). However, there was no evidence for a difference between the genders and these barriers to care (*p* = 0.19 and *p* = 0.30, respectively).

Lack of social support (36 % of women vs 27 % of men; *p* = 0.17), as well as distance to facilities capable of providing essential surgical care (87 % of women vs 81 % of men; *p* = 0.25) were also commonly reported barriers to care.

More women reported having no one to accompany them for surgery and post-operative care compared with men (30; 20 % vs 19; 11 %, *p* = 0.03). Thirty-eight women (26 %) reported not being able to access surgical care because they were not the decision maker in the household and 49 (33 %) reported inordinately long waiting times to get surgical care after referral. The corresponding proportions for men were 16 % and 22 %, respectively (*p* = 0.03). There was a significant difference in the proportion of women (20; 14 %) reporting fear of anesthesia as a barrier to accepting surgical care compared with men (6; 4 %, *p* = 0.001).

### Female-specific barriers not captured by the tool

Other barriers offered by women for being unable to access timely surgical care generally fell under the acceptability dimension and included: not trusting healthcare personnel to keep their condition and/or surgery confidential and not wanting to expose themselves for examinations in consulting rooms that are often not private enough. The rest were fear of being considered a witch after the community learns that she has a gynecological problem and fear she would be ridiculed for seeking surgical consultation or care in order to get pregnant.

## Discussion

This study aimed to define barriers to surgical care that women face in five communities in the two northernmost regions of Ghana. While significant barriers to essential surgical care affected both genders, women more often reported barriers than men. This was evidenced by the lower acceptability and accessibility dimension scores among women compared with men, indicating more significant barriers in these areas. Lack of social support, inability to navigate the healthcare system, fear of anesthesia and inadequate privacy were also frequently reported. The disparate indices between genders within the same geographic region highlights the importance of systematically identifying and addressing barriers to care at the community level to improve the uptake of essential surgical care. Despite these differences, there were other barriers for which there was little or no difference between genders. These included some level of misunderstanding about their conditions (e.g. not knowing that a surgical condition can be treated by surgery, seeking cure from traditional healers); structural barriers (e.g., not having personnel capable of performing more than minor procedures, as well as travel distance); and inability to afford essential surgical care.

In their systematic review, Grimes et al. documented barriers to surgical care in LMICs. Their search retrieved reports describing barriers to care for a number of surgical specialties, including ophthalmology, emergency care, and burns. However, there was not a report describing barriers to essential general surgical care among women in LMICs [9]. Nonetheless, the retrieved reports found that distance to health facilities, lack of awareness about the need for surgical intervention for certain conditions, and perceived poor quality of surgical services were among common barriers requiring redress. In addition to these barriers, this study identifies other barriers to general surgical care that include: the influence of other family members on decision-making, fear of anesthesia and/or surgical care, and mistrust of surgical care (i.e. not appreciating the potential benefit and/or safety of surgical care), which should be assessed when trying to develop interventions to improve the uptake of essential surgery in LMICs.

The value of systematically assessing barriers to care at the community level is that, with consideration of local contexts, the results can inform interventions that may improve access to surgical care. For instance, women were more likely to report not having sufficient social support at home or during their hospital stay, which prevented care seeking. In LMICs, surgery is often only available at referral centers, far from patients’ homes [17]; the median travel time to health facilities in our study population was 60 min, though it was up to 8 h for some women. Additionally, hospital resource deficiencies mean that many family members typically shoulder patient care responsibilities (e.g. cleaning, feeding and washing). When women don’t have someone to accompany them for surgery, they do not present for care and incur preventable disability or even death [8, 18]. To overcome this barrier, facilities could employ non-healthcare personnel to provide these services or establish a volunteer service, which has been successful in some high-income countries [19]. This barrier could also be overcome by providing more frequent surgical outreaches or temporarily posting a provider capable of essential surgical care in these particularly deprived areas, which might prevent women from having to travel beyond their social support network. Thus, the growing backlog of conditions that incur disability from untreated elective essential surgical conditions might decrease.

Fear of anesthesia was commonly reported as a reason for not seeking surgical care among women. Given similar findings from Turkey, Nigeria and India, this finding is not unique to Ghana [20–22]. Currently, patients scheduled for elective surgery at these facilities are asked to present on the day of their operation and do not get sensitized to the perioperative process beforehand. As a result, patients may have considerable apprehension about anesthesia care. In high-income countries, many surgical services have included a brief, routine pre-anesthesia consultation prior to planned surgery to set expectations and allay fears regarding the perioperative process. Such an encounter might improve the perception of anesthesia among women in communities that are particularly fearful. Further, efforts to incorporate knowledge about anesthesia and surgical care into community-based health promotion initiatives might also be considered.

Privacy and confidentiality are central tenets of medical ethics. However, they have not been prioritized in many LMIC healthcare facilities, which operate above capacity to meet patient demand. As a result, consulting rooms, pre-operative holding areas and wards are often not private enough. While it has been suggested that healthcare privacy is not as valued by LMIC patients as those in high-income countries, this is certainly not the case as our results demonstrate and others have shown [23–27]. All efforts should be made to respect patient privacy and confidentiality by designating private changing areas, draping patients appropriately, using privacy screens and keeping conversations and records confidential [27]. These issues have been identified by studies on patient satisfaction after receiving maternal care services in LMICs [25, 28]. Inadequate privacy during antenatal checkup, lack of confidentiality and being exposed during examination or during delivery were all significant predictors of low satisfaction scores, and in turn, poor compliance and retention [25, 29]. Essential surgical care services might consider incorporating monitoring and evaluation of privacy and confidentiality into their mandate, which may reduce these barriers to care in the long run.

Several limitations are worth consideration when interpreting these findings. First, the barriers offered by respondents who presented for surgery during the outreach might be different from those that did not present for care. Hospital-based assessments of barriers to surgical care are likely associated with different barriers than those identified by community-based studies [30, 31]. Furthermore, selection bias may also exist as a result of differential response to mobilization efforts between men and women. However, participants in this study were aggressively mobilized from their communities and reported long durations of disease. This group therefore likely represents an adequate intermediate between hospital-based and community-based populations and still provides important barriers that require redress. Second, use of healthcare workers as research team members might have introduced interviewer bias. However, healthcare workers had expertise in structured interpersonal interaction that was useful for rapidly developing the skills required for interviewing. Additionally, they understood the surgical care context within which this study was conducted. These valuable qualities of healthcare workers might moderate the potential interviewer bias rather than create it. Lastly, the assessment tool was designed to identify individual- and community-level barriers to care. Therefore, other important governance and policy barriers may exist that were not measured [8]. However, such issues would likely be reflected, at least in part, by the barriers we identified. Despite these limitations, the results from this study allow reasonable conclusions to be drawn about the barriers to essential surgical care experienced by women in the two northernmost regions of Ghana.

## Conclusion

Women in the two northernmost regions of Ghana have more barriers to essential surgical care than their male counterparts. Specific barriers that could be addressed to improve surgical care for people living in these communities, especially among women, include: a lack of social support during hospital stays, fear of anesthesia and inadequate privacy. To improve these barriers, hospitals might consider employing staff or recruiting volunteers to assist women during their hospital stay. Next, there is the need to provide community-specific education to sensitize potentially vulnerable populations about the safety and benefits of essential surgical care where appropriate. Additionally, pre-anesthesia consultations for patients in need of elective surgical care might reduce fears of the perioperative process. Lastly, health facilities should provide a private and confidential environment for all patients, so that they feel respected and safe. In turn, more people with potentially correctable surgical conditions might present for care. Together, such interventions may significantly improve the uptake of elective essential surgical care in Ghana, as well as other LMICs.

## Abbreviations

AMOG, ApriDec Medical Outreach Group; DCP-3, Disease Control Priorities, third edition; LMIC, low- and middle-income countries; NGO, non-governmental organization.

## Additional file

Additional file 1: Supplementary material. (DOCX 114 kb)

## References

[1] Alkire BC, Raykar NP, Shrime MG, Weiser TG, Bickler SW, Rose JA, Nutt CT, Greenberg SL, Kotagal M, Riesel JN (2015). Global access to surgical care: a modelling study. Lancet Global Health.

[2] Mock CN, Donkor P, Gawande A, Jamison DT, Kruk ME, Debas HT (2015). Essential surgery: key messages from Disease Control Priorities, 3rd edition. Lancet.

[3] Groen RS, Samai M, Stewart KA, Cassidy LD, Kamara TB, Yambasu SE, Kingham TP, Kushner AL (2012). Untreated surgical conditions in Sierra Leone: a cluster randomised, cross-sectional, countrywide survey. Lancet.

[4] Petroze RT, Groen RS, Niyonkuru F, Mallory M, Ntaganda E, Joharifard S, V Estimating operative disease prevalence in a low-income country: results of a nationwide population survey in Rwanda. Surgery. 2013;153(4):457–64. 10.1016/j.surg.2012.10.001 23253378

[5] Stewart BT, Pathak J, Gupta S, Shrestha S, Groen RS, Nwomeh BC, Kushner AL, McIntyre T (2015). An estimate of hernia prevalence in Nepal from a countrywide community survey. Int J Surg.

[6] Carlson LC, Lin JA, Ameh EA, Mulwafu W, Donkor P, Derbew M, Rodas E, Mkandawire NC, Dhanaraj M, Yangni-Angate H (2015). Moving from data collection to application: a systematic literature review of surgical capacity assessments and their applications. World J Surg.

[7] Meara JG, Leather AJ, Hagander L, Alkire BC, Alonso N, Ameh EA, Bickler SW, Conteh L, Dare AJ, Davies J et al. Global Surgery 2030: evidence and solutions for achieving health, welfare, and economic development. Surgery. 2015;158:3–6. 10.1016/j.surg.2015.04.011 25987187

[8] Irfan FB, Irfan BB, Spiegel DA (2012). Barriers to accessing surgical care in Pakistan: healthcare barrier model and quantitative systematic review. J Surg Res.

[9] Grimes CE, Bowman KG, Dodgion CM, Lavy CB (2011). Systematic review of barriers to surgical care in low-income and middle-income countries. World J Surg.

[10] Stewart BT, Gyedu A, Abantanga F, Abdulai AR, Boakye G, Kushner A (2015). Barriers to essential surgical care in low- and middle-income countries: a pilot study of a comprehensive assessment tool in Ghana. World J Surg.

[11] Wong EG, Gupta S, Deckelbaum DL, Razek T, Kamara TB, Nwomeh BC, Haider AH, Kushner AL (2014). The International Assessment of Capacity for Trauma (INTACT): an index for trauma capacity in low-income countries. J Surg Res.

[12] Choo S, Perry H, Hesse AA, Abantanga F, Sory E, Osen H, Fleischer-Djoleto C, Moresky R, McCord CW, Cherian M (2010). Assessment of capacity for surgery, obstetrics and anaesthesia in 17 Ghanaian hospitals using a WHO assessment tool. Tropical Med Int Health.

[13] Choo S, Perry H, Hesse AA, Abantanga F, Sory E, Osen H, Fleischer-Djoleto C, Moresky R, McCord CW, Cherian M et al: Surgical training and experience of medical officers in Ghana’s district hospitals. Acad Med. 2011;86(4):529–33. 10.1097/ACM.0b013e31820dc471 21346502

[14] Bradley EH, Curry LA, Devers KJ (2007). Qualitative data analysis for health services research: developing taxonomy, themes, and theory. Health Serv Res.

[15] Bornstein MH, Britto PR, Nonoyama-Tarumi Y, Ota Y, Petrovic O, Putnick DL (2012). Child development in developing countries: introduction and methods. Child Dev.

[16] Myers ND, Ahn S, Jin Y (2011). Sample size and power estimates for a confirmatory factor analytic model in exercise and sport: a Monte Carlo approach. Res Q Exerc Sport.

[17] Ntirenganya F, Petroze RT, Kamara TB, Groen RS, Kushner AL, Kyamanywa P, Calland JF, Kingham TP (2014). Prevalence of breast masses and barriers to care: results from a population-based survey in Rwanda and Sierra Leone. J Surg Oncol.

[18] Filippi V, Richard F, Lange I, Ouattara F (2009). Identifying barriers from home to the appropriate hospital through near-miss audits in developing countries. Best Pract Res Clin Obstet Gynaecol.

[19] Volunteer services, Moran Surgical Center. Utah, USA: Salt Lake City. [http://healthcare.utah.edu/hospital/customer-service/volunteer/opportunities/moran_surgery_center.php]. Accessed 1 Sept 2015.

[20] Gurunathan U, Jacob R (2004). The public’s perception of anaesthesiologists-Indian Attitudes. Indian J Anaesth.

[21] Osinaike BB, Dairo MD, Oyebamiji EO, Odesanya JO, Tanimowo A (2007). Attitude of general public to risks associated with anaesthesia. East Afr J Public Health.

[22] Sagun A, Birbicer H, Yapici G (2013). Patients’, who applied to the anesthesia clinic, perceptions and knowledge about anesthesia in Turkiye. Saudi J Anaesthesia.

[23] Das P, Basu M, Tikadar T, Biswas G, Mridha P, Pal R (2010). Client satisfaction on maternal and child health services in rural bengal. Indian J Community Med.

[24] Jallow IK, Chou YJ, Liu TL, Huang N (2012). Women’s perception of antenatal care services in public and private clinics in the Gambia. Int J Qual Health Care.

[25] Nigenda G, Langer A, Kuchaisit C, Romero M, Rojas G, Al-Osimy M, Villar J, Garcia J, Al-Mazrou Y, Ba'aqeel H (2003). Womens’ opinions on antenatal care in developing countries: results of a study in Cuba, Thailand, Saudi Arabia and Argentina. BMC Public Health.

[26] Simbar M, Nahidi F, Dolatian M, Akbarzadeh A (2012). Assessment of quality of prenatal care in Shahid Beheshti Medical Science University centers. Int J Health Care Qual Assur.

[27] Srivastava A, Avan BI, Rajbangshi P, Bhattacharyya S (2015). Determinants of women’s satisfaction with maternal health care: a review of literature from developing countries. BMC Pregnancy Childbirth.

[28] Tetui M, Ekirapa EK, Bua J, Mutebi A, Tweheyo R, Waiswa P (2012). Quality of Antenatal care services in eastern Uganda: implications for interventions. Pan Afr Med J.

[29] Balogun OR (2007). Patients perception of quality of antenatal service in four selected private health facilities in Ilorin, Kwara state of Nigeria. Niger Med Pract.

[30] Bickler S, Ozgediz D, Gosselin R, Weiser T, Spiegel D, Hsia R, Dunbar P, McQueen K, Jamison D (2010). Key concepts for estimating the burden of surgical conditions and the unmet need for surgical care. World J Surg.

[31] Groen RS, Samai M, Petroze RT, Kamara TB, Yambasu SE, Calland JF, Kingham TP, Guterbock TM, Choo B, Kushner AL (2012). Pilot testing of a population-based surgical survey tool in Sierra Leone. World J Surg.

