# Supplementary material for: Barriers to essential surgical care experienced by women in the two northernmost regions of Ghana: a cross-sectional survey
Source: BMC Womens Health. 2016 May 26;16:27. doi: 10.1186/s12905-016-0308-4 (PMC4882854; doi:10.1186/s12905-016-0308-4)
Supplement: Supplementary file 1 — Supplementary material. (DOCX 114 kb) [file 12905_2016_308_MOESM1_ESM.docx]

**Supplementary material legend**

Supplementary material 1: Barriers to surgical care assessment tool

Supplementary material 2: Confirmatory factor analysis path diagram with standardize factor loading values for each question and dimension of the barriers to surgical care assessment tool

Supplementary material 1: Barriers to surgical care assessment tool

| A3 Tool for Assessing Barriers to Surgical Care | | | | | | | | |
| --- | --- | --- | --- | --- | --- | --- | --- | --- |
|  |  |  |  |  |  |  |  |  |
|  | | | | | | | | |
| 1. | Country |  |  |  |  |  |  |  |
| 2. | Project or community name |  |  |  |  |  |  |  |
| 3. | Name of interviewer |  |  |  |  |  |  |  |
| 4. | Phone number of interviewer |  |  |  |  |  |  |  |
|  |  |  |  |  |  |  |  |  |
| 5. | What surgery are you having performed? | Hernia or hydrocele | Thyroid | Skin or soft-tissue | Breast | Gynecologic | Orthopedic | Other |
| 6. | What type of hospital is performing the surgery? | District | Regional | Tertiary | Teaching | Mission | NGO | Private |
| 7. | How long did you travel to get to the hospital from your home (including time waiting for transport)? | min | hrs | days |  |  |  |  |
| 8. | Is this hospital the closest one capable of performing your surgery to where you live? | Yes | No |  |  |  |  |  |
| 9. | If no to 7, what kind of hospital capable of performing your surgery is closer to where you live? | District | Regional | Tertiary | Teaching | Mission | NGO | Private |
| 10. | If no to 7, why did you bypass a closer hospital capable of performing your surgery? |  | | | | | | |
|  |  |  |  |  |  |  |  |  |
| 11. | How old are you? | yrs |  |  |  |  |  |  |
| 12. | Gender | Male | Female |  |  |  |  |  |
| 13. | What is the most school you have completed? | None | Primary | Secondary | College | More |  |  |
| 14. | What religion do you practice? | Christianity | Islam | Traditional | Hindu | Buddhism | Judaism | Other |
| 15. | Have you ever had the surgery you are having today? | Yes | No |  |  |  |  |  |
| 16. | Have you previously had another type of surgery? (if no skip to 17) | Yes | No |  |  |  |  |  |
| 17. | If yes to either 14 or 15 did you ever have a complication? | Yes | No |  |  |  |  |  |
| 18. | If yes to 16, what are the details of most serious complication that occurred? | No treatment needed | Medicines given | Procedure or reoperation | Still in need of more care |  |  |  |
| 19. | How long have you had this problem needing surgery? | Days | Weeks | Months | Years | Since birth |  |  |
| 20. | If your problem has symptoms, how long have you had symptoms for? | Never | Days | Weeks | Months | Years | Since birth |  |

|  |  |  |  |
| --- | --- | --- | --- |
|  |  | **Contributing reasons** | |
|  |  | True/Yes (1) | False/No (0) |
|  | Which of the following **reasons** **for not having surgery for your problem sooner** apply to you? Is it because: READ EACH QUESTION. (more than one answer may apply) |  |  |
|  | **Acceptability (culture and understanding) [18]** |  |  |
| 21. | …you are afraid of anesthesia? |  |  |
| 22. | …you are afraid of surgery? |  |  |
| 23. | …you are afraid of pain after surgery? |  |  |
| 24. | …you are afraid of a specific surgeon or anesthestist? |  |  |
| 25. | …you are afraid that surgery will impair your sexual function? |  |  |
| 26. | ...there are rumors or do you know of complications from another person's surgery? |  |  |
| 27. | …you are not the decision maker in the household? |  |  |
| 28. | …you are too young or too old for surgery? |  |  |
| 29. | …surgery is not considered appropriate for your social role (i.e. beggar, class)? |  |  |
| 30. | …you know most people with your problem need surgery, but didn't think yours needed surgery? |  |  |
| 31. | …you didn't feel informed enough about the procedure or recovery to undergo surgery? |  |  |
| 32. | …you have been treated for problem by a traditional healer instead of having surgery? |  |  |
| 33. | …you were not worried that your problem could become an emergency? |  |  |
| 34. | …you were told that your problem didn't require surgery by someone in the community? |  |  |
| 35. | …your problem did not impair your daily work but now it does (but not pain)? |  |  |
| 36. | …your problem did not cause pain that impairs your daily work but now it does? |  |  |
| 37. | …your problem did not impair your sexual function but now it does? |  |  |
| 38. | ...you feel you will be disfigured or not be a whole person after having surgery? |  |  |
|  | **Sub-total** |  | |
|  | **Multiply sub-total by 1.44** |  | |

|  |  |  |  |
| --- | --- | --- | --- |
|  |  | **Contributing reasons** | |
|  |  | True/Yes (1) | False/No (0) |
|  | Which of the following **reasons** **for not having surgery for your problem sooner** apply to you? Is it because: READ EACH QUESTION. (more than one answer may apply) |  |  |
|  | **Affordability (cost and social support) [4]** |  |  |
| 39. | …the costs of hospital/doctor fees, transport, food or a carer for getting surgery for your problem were too much for you to afford (direct costs)? |  |  |
| 40. | …you would have lost money from not working while you were away getting surgery for your problem (indirect costs)? |  |  |
| 41. | …you had no one to help care for your family, farm, work or home while you were gone? |  |  |
| 42. | …no one would accompany you for surgery? |  |  |
|  | **Sub-total** |  | |
|  | **Multiply sub-total by 6.5** |  | |
|  | **Accessibility (healthcare and structural) [4]** |  |  |
| 43. | …a healthcare worker told you that you did not have a problem that needed surgery? |  |  |
| 44. | …the distance was too far for you to travel? |  |  |
| 45. | …it was too hard or took a long time for you to be referred to a hospital that could provide the surgery? |  |  |
| 46. | …there was a long waiting time between being told you needed surgery and having it? |  |  |
|  | **Sub-total** |  | |
|  | **Multiply sub-total by 6.5** |  | |
|  |  |  |  |
|  | **Total** |  | |
|  | **Divide total by 84 (round to nearest whole number)** |  | |
|  | **Multiply divided total by 10 to get index** |  | |
| 47. | What other reasons do you have for not previously having surgery for your problem? |  |  |
|  |  |  |  |
|  |  |  |  |
|  |  |  |  |
|  |  |  |  |
|  |  |  |  |
|  |  |  |  |

48. ASK TO ONLY FEMALE RESPONDENTS

Are there any other specific reasons that have prevented you from receiving surgical care, because you are a woman?

Supplementary material 2: Confirmatory factor analysis path diagram with standardize factor loading values for each question and dimension of the barriers to surgical care assessment tool

*p<0.05; Overall root mean standard error of approximation (RMSEA) <0.01; overall coefficient of determination (CD) 0.76; Acceptability RMSEA 0.07; CD 0.85; Affordability RMSEA 0.08; Affordability CD 0.66; Accessibility RMSEA 0.04; Accessibility CD 0.55

0.48*

0.16*

0.86*

0.42*

0.68*

0.83*

0.26*

0.45*

0.71*

0.21*

0.10

0.13*

0.46*

0.46*

0.28*

0.23*

0.23*

0.06

0.04

0.23*

0.14*

0.20*

0.47*

0.75*

0.50*

0.29*

0.02

0.28*

0.57*

0.30*
